# Bleb failure and intraocular pressure rise following Nd: Yag laser capsulotomy

**DOI:** 10.1186/s12886-017-0408-4

**Published:** 2017-02-22

**Authors:** Andreas Diagourtas, Petros Petrou, Ilias Georgalas, Kostantinos Oikonomakis, Panagiotis Giannakouras, Athanasios Vergados, Dimitrios Papaconstantinou

**Affiliations:** Athens A’ University Eye Clinic, “Georgios Gennimatas” General Hospital, Athens, Greece

**Keywords:** Case series, Capsulotomy, Drainage device, Intraocular pressure, Trabeculectomy

## Abstract

**Background:**

To report the negative effect of Nd: Yag (Neodymium-doped: Yttrium Aluminium Garnet) laser capsulotomy on the intraocular pressure (IOP) and the trabeculectomy bleb integrity, in a small series of eyes, both trabeculectomised and pseudophakic, following the laser application for the management of posterior capsular opacification (PCO).

**Methods:**

This is a retrospective, non-comparative interventional case series study, in which 20 trabeculectomised and pseudophakic eyes from 15 patients, with otherwise well functioning blebs, were presented with uncontrolled IOP, in a variable distance of time following the application of YAG laser capsulotomy. Student paired *t*-test confirmed a statistically significant difference (*P* < 0.05) between IOP before Nd: YAG laser capsulotomy (16 mmHg ± 3 mmHg) and the respective one, 2 to 6 months after Nd: Yag capsulotomy (34.5 ± 11 mmHg).

**Results:**

All of the cases failed to respond to conservative treatment and were successfully managed with the implantation of Ahmed drainage devices. All patients showed flat filtering bleb and uncontrolled IOP (34.5 ± 11 mmHg), under maximum topical treatment, in a period of 2 to 6 months following Nd: YAG laser caspulotomy. The implantation of Ahmed valve proved to be effective treatment for these patients (IOP < 21 mmHg).

**Conclusions:**

Although Nd: Yag laser capsulotomy is considered a safe surgical procedure and usually is done without second thought, in this series of eyes, it is postulated that it may be responsible for the deregulation of the filtering bleb and subsequent loss of IOP control. We consider that laser capsulotomy should be performed with caution, especially in eyes with previous trabeculectomy. Also close monitoring of the intraocular pressure and assessment of eventual bleb morphology variations in the follow-up period is mandatory. Further studies are needed in order to confirm our findings.

## Background

Posterior capsule opacification (PCO) represents one of the commonest delayed post-operative complications following extracapsular cataract extraction and phacoemulsification. According to literature reports, the incidence of PCO after cataract surgery and intraocular lens implantation varies between 15 and 43% [1–3].

Although Nd: YAG laser (Neodymium-doped: Yttrium Aluminium Garnet laser) posterior capsulotomy is a common procedure for the management of PCO, it has been associated with several complications, some of which severe.

Short term elevation in the intraocular pressure (IOP spikes) following Nd: YAG laser capsulotomy has been well documented [4, 5] and several studies have shown the long term effect of this procedure on IOP, in non glaucomatous patients [6–8].

However, only few published studies evaluate the long term changes in IOP, after Nd: YAG laser capsulotomy, in eyes with glaucoma that have previously undergone trabeculectomy [9, 10].

The purpose of the present study is to report the effect of Nd: Yag laser capsulotomy on IOP control and trabeculectomy bleb integrity in a series of otherwise well-controlled glaucomatous patients, and to document the inadequate control of the IOP with medical treatment and the success of further surgical management.

## Methods

The present study represents a retrospective, non-comparative, interventional, case series. The study adheres to the tenets of the Declaration of Helsinki and was performed after the permission of the institutional ethics committee of “G.Gennimatas” General Hospital. Patients were informed about the study procedure and eventual ocular image publication and provided written, informed consent.

We reviewed the medical records of 15 patients (20 eyes) who were referred to our glaucoma department for management of uncontrolled IOP, 2 to 6 months after Nd; YAG laser capsulotomy (Table 1).

**Table 1:** Patients’ characteristics

Patients/Eyes	15/20
Age (yrs)	
Mean (±SD)	56.2 ± 24.9
Range (years)	14–77
Median	49
Male	8 (53%)
Female	7 (47%)
Type of glaucoma (Eyes)	
POAG	11 (55%)
PXG	5 (25%)
UG	2 (10%)
CACG	2 (10%)

*SD* standard deviation, *IOP* intraocular pressure, *POAG* primary-open angle glaucoma, *PXG* pseudoexfoliative glaucoma, *UG* uveitic glaucoma, *CACG* chronic angle closure glaucoma, *BCVA* best corrected visual acuity

All eyes had primarily undergone standard trabeculectomy and cataract extraction followed (period between trabeculectomy and phacoemulsification ranged between 1 and 7 years).

Trabeculectomy was performed by the same surgeon (D.P.). Following peribulbar anaesthesia with lidocaine 1%, the eye was prepared and draped. A 7-0 silk suture was placed half thickness in the superior cornea as a traction suture. All operations were performed with fornix-based conjunctival flaps centered at 12 o’ clock and a rectangular scleral flap of approximately 3 × 5 mm.

A knife operated sclerectomy (1 × 2 mm) was followed by a basic-peripheral iridectomy. After the verification of the ostium patency, the scleral flap was closed with 2 10-0 nylon sutures, one in each angle. At last, tenon’s and conjunctiva were advanced and sutured at the level of the limbus usually with 2 to 3 10-0 nylon sutures.

Postoperatively, a topical fixed combination of chloramphenicol and dexamethasone six times daily for 4 weeks followed by gradual tapering was used.

One to 7 years after the trabeculectomy the eyes underwent cataract extraction, phacoemulsification (*n* = 16) or extracapsular cataract extraction (*n* = 4).

All cases were uncomplicated, with in-the-bag intraocular lens implantation. Subsequently, they developed posterior capsular opacification and underwent Nd; YAG laser capsulotomy.

Before the laser procedure their IOPs were within our estimated target pressure, with either the use of topical medication (three eyes) or without any (the rest).

Nd; YAG laser capsulotomy was performed in all the eyes by the same laser device, but by various operators and usually with variable laser settings. Nontheless, the amount of total energy never exceeded the 10 mJ. A capsulotomy contact lens was not used in any of the cases. Apraclonidine was administered in all the eyes immediately on completion of the Nd: YAG laser posterior capsulotomy to minimize a postoperative intraocular pressure spike and patient’s IOP was measured one hour after the laser procedure.

A short course of topical steroids was used following the procedure and follow-up appointment was scheduled for a week after.

Data about the pre and post-operative best-corrected visual acuity and clinical examination (including gonioscopy and fundus examination), pre and post-operative IOP (Goldmann applanation tonometry) and bleb integrity as well as the pre and post-operative medication were collected and analysed.

## Results

Twenty eyes of fifteen patients were included in the analysis. The mean age of the patients was 56.2 ± 24.9 (range 14–77 years) and there was no gender preponderance (eight males, seven females). Seven patients (11 eyes) suffered from chronic open angle glaucoma, four patients (five eyes) had pseudoexfoliative glaucoma, two patients (two eyes) had chronic angle closure glaucoma and two patients (two eyes) had uveitic glaucoma (Table 1).

The mean IOP before Nd: YAG laser capsulotomy was 16 mmHg (±3 mmHg) without any glaucoma treatment in 17 eyes. Three eyes required the administration of a single medication for IOP control.

Nd; YAG laser capsulotomy was performed between 12 and 42 months after the cataract extraction and the intraocular lens (IOL) implantation. Mean IOP 2 to 6 months after Nd; YAG laser capsulotomy was 34.5 ± 11 mmHg (student paired *t*-test, *P* < 0.05 compared to the pre-yag laser IOP), with all patients under maximum topical treatment. Furthermore, in four patients oral acetazolamide (250 mg or 125 mg three times a day) was administered in order to achieve adequate IOP control.

All patients underwent Ahmed valve implantation with a mean follow-up of 8.5 months during which IOP was adequately controlled (<21 mmHg) and either topical or systemic antiglaucomatous medication was required.

## Discussion

In the present study, a series of patients with well functioning bleb and controlled IOP after trabeculectomy and secondary cataract extraction demonstrated loss of the bleb integrity and IOP control, following Nd: YAG laser capsulotomy for PCO. Thus, further surgical treatment (Ahmed valve implantation) was required in order to control the IOP.

The effect of Nd: YAG laser capsulotomy on the IOP either during the short post-laser period or in the long term is well documented in the literature, both in the general population, as well as in glaucomatous patients. Several studies have shown that there is high incidence of IOP rise in the immediate postoperative period following Nd: YAG laser capsulotomy [4, 6, 11]. The IOP elevation initiates within hours following the procedure and the maximum IOP values are recorded within the first 2 days [11–13]. The incidence of newly diagnosed glaucoma due to sustained IOP elevation after Nd: YAG laser capsulotomy has been reported to be up to 6% [11, 13, 14]. Furthermore, pre-existing glaucoma is a well known risk factor for transient IOP elevations after Nd: YAG laser capsulotomy [10, 11, 13]. Several studies have documented the protective effect of hypotensive pre-treatment in glaucomatous patients undergoing laser capsulotomy [15].

The underlying mechanism of this rise in IOP following Nd: YAG laser capsulotomy remains unclear. Pathophysiological hypotheses include effects on the ciliary body function caused by laser shock waves, a neurohumoral increase in the IOP, structural effects of laser energy on sodium hyaluronate and finally, mechanical blockage of the trabecular meshwork by various debris, such as fragments from the disrupted posterior capsule or vitreous [4, 10].

However, there are very few studies in the literature evaluating the effect of Nd: YAG laser capsulotomy on the IOP of pseudophakic patients that have previously undergone well functioning filtering procedures. Zeyen et al. examined 20 patients that had undergone combined cataract–glaucoma procedure followed by Nd: YAG capsulotomy, with 3 months interval and concluded that the capsulotomy does not significantly alter the bleb function or the number of glaucoma medications used after the procedure [9]. Recently, Lin et al. published their results from the follow up of 69 glaucoma patients that underwent Nd: YAG laser capsulotomy. Among them, 42 patients had previously undergone combined cataract and glaucoma procedure. The authors concluded that the loss of baseline IOP control after Nd: YAG laser capsulotomy is common in glaucoma patients, however it is unclear whether this increase in the IOP is related directly to the laser procedure or whether this is an independent progression of the patient’s glaucoma [10].

In all our cases, patients demonstrated loss of IOP control, in spite of maximum topical treatment, a few months after Nd: YAG laser capsulotomy. On slit lamp examination the appearance of the bleb was mostly flat. All eyes had previously undergone trabeculectomy with well formed and functioning blebs and IOP was adequately controlled in all follow-up visits prior to the laser procedure. It is highly unlikely that this loss of IOP control, documented after Nd: YAG capsulotomy, is a natural progression of glaucoma disease and not directly related to the procedure.

It is postulated that the underlying mechanism of this complication could be the ab interno mechanical blockage of the filtering bleb. Thus, debris from the disrupted posterior capsule or liquefied vitreous, following capsulotomy, is considered to pass through the site of the iridectomy, obstructing the sclerostomy In addition, the fact that in all the affected eyes the size of the anterior capsulotomy was quite large should be taken into consideration, as this event probably facilitated the transportation of debris or vitreous from the posterior to the anterior chamber (AC), which subsequently blocked the filtering bleb [16].

The above taken into consideration, our team proceeded to the implantation of an Ahmed valve in all eyes. The decision for the use of a drainage device was based on the fact that the inner aperture of the tube could be placed deep inside the anterior chamber, transferring the acting position of the sclerostomy away from the corneo-scleral angle, thus reducing the chance of occlusion by vitreous or debris.

Our study has some obvious limitations. It is retrospective with a limited number of patients without a control group. Nonetheless, to the best of our knowledge it represents one of the very few reports addressing the influence of Nd: YAG laser capsulotomy on IOP in trabeculectomised eyes and suggesting the mechanical blockage of the sclerostomy as a possible pathophysiological mechanism. More extensive longitudinal and prospective studies are needed to verify our hypothesis. This will facilitate patient counselling.

## Conclusion

In conclusion, it is postulated that Nd: Yag capsulotomy may be responsible for the deregulation of the trabeculectomy bleb and subsequent loss of IOP control in previously trabeculectomized eyes undergoing management of PCO. Further comparative studies are needed in order to confirm our findings.

## Abbreviations

AC: Anterior chamber
IOL: Intraocular lens
IOP: Intraocular pressure
Nd: Yag: Neodymium-doped: Yttrium Aluminium Garnet
PCO: Posterior capsular opacification
